# Starting from scratch: a workflow for building truly novel proteins

**DOI:** 10.1093/synbio/ysab005

**Published:** 2021-01-30

**Authors:** Pablo Cárdenas

**Affiliations:** Department of Biological Engineering, Massachusetts Institute of Technology, Cambridge, MA 02139, USA

In its early days, synthetic biology was often defined as repurposing existing biological parts for new applications. More recently, we have seen work that pushes the boundaries of the field past repurposing and into the design of truly novel biological parts. To date, most attempts at designing complex, non-structural protein functions have hinged on grafting protein motifs with known functions onto synthetic protein scaffolds. This ‘top-down’ approach to synthetic protein design unfortunately depends on the structural compatibility of the functional sites with rigid scaffolds used.

A study recently published in *Nature Chemical Biology* describes an alternative ‘bottom-up’ approach in which structural elements are created *de novo* to support the functional elements in whatever conformation they are specified in (1). The multi-institutional team led by Bruno Correira’s lab at École Polytechnique Fédérale de Lausanne demonstrated the efficacy of their novel approach by building tunable biosensors for epitope-specific antibodies and dual-specific ligands for synthetic cell receptors.

To achieve this ‘bottom-up’ design, the authors used TopoBuilder (2), a previously published computational tool, to generate three-dimensional ‘sketches’ of a given protein of interest with specific functional motifs in their idealized conformations. With the help of another software tool, Rosetta FunFoldDes (3), the authors created and simulated tens of thousands of possible designs fulfilling the design criteria, which were filtered by favorable thermodynamic predictions for stability and folding. A combinatorial library of up to 10 million variants was built from elements of the best designs. The libraries were then screened by binding affinity to the desired target(s) and protease digestion using yeast surface display, and the best protein variants were selected by sequencing the output. Finally, the structure and behavior of the final products were determined using a variety of different physical and chemical assays.

The authors used their workflow to design a biosensor based on bioluminescence resonant energy transfer (4) to sense antibodies with affinities for a single, specific epitope found in respiratory syncytial virus and metapneumovirus, two respiratory pathogens. The novel design pipeline made it possible to present the single epitope in scaffolds with different binding affinities to the target antibody. This, in turn, allowed for tuning the biosensor’s response.

Furthermore, the workflow was used to create a synthetic ligand capable of binding to two different, previously created synthetic mammalian signal receptors, which trigger expression of a reporter gene (5). The authors demonstrated the ligand simultaneously bound its two distinct targets by showing it only triggered output signal when both types of receptor were present in a cell.

The work presented by Yang *et al*. is exciting for its practically universal applicability in biological research. Applications could range from building self-assembling, functionalized biomaterials with complex structure to modulating immune responses using synthetic proteins tailor-made to fit receptor complexes. It remains to be seen whether further work can do away with the extensive library screening still required, or how more complex protein functions such as reaction catalysis or allostery fare with these techniques. Nevertheless, bottom-up *de novo* protein design in one form or another seems poised to become a bioengineering staple in the future.

*Conflict of interest statement*. None declared.
